# Side channel analysis based on feature fusion network

**DOI:** 10.1371/journal.pone.0274616

**Published:** 2022-10-17

**Authors:** Feng Ni, Junnian Wang, Jialin Tang, Wenjun Yu, Ruihan Xu

**Affiliations:** 1 School of Physics and Electronics, Hunan University of Science and Technology, Xiangtan, China; 2 Hunan Provincial Key Laboratory of Intelligent Sensors and Advanced Sensor Materials, Xiangtan, Hunan, China; University of Belgrade Faculty of Organisational Sciences: Univerzitet u Beogradu Fakultet organizacionih nauka, SERBIA

## Abstract

Various physical information can be leaked while the encryption algorithm is running in the device. Side-channel analysis exploits these leakages to recover keys. Due to the sensitivity of deep learning to the data features, the efficiency and accuracy of side channel analysis are effectively improved with the application of deep learning algorithms. However, a considerable part of existing reserches are based on traditional neural networks. The effectiveness of key recovery is improved by increasing the size of the network. However, the computational complexity of the algorithm increases accordingly. Problems such as overfitting, low training efficiency, and low feature extraction ability also occur. In this paper, we construct an improved lightweight convolutional neural network based on the feature fusion network. The new network and the traditional neural networks are respectively applied to the side-channel analysis for comparative experiments. The results show that the new network has faster convergence, better robustness and higher accuracy. No overfitting has occurred. A heatmap visualization method was introduced for analysis. The new network has higher heat value and more concentration in the key interval. Side-channel analysis based on feature fusion network has better performance, compared with the ones based on traditional neural networks.

## Introduction

Cryptography is an important foundation of modern information security technology. But just because an encryption algorithm is secure by design does not mean that it can be guaranteed to be equally secure when implemented in hardware. Physical information such as time [1], power consumption [2] and electromagnetic radiation [3] can be compromised when cryptographic algorithms are running on cryptographic hardware devices. These physical information can be exploited to perform a Side Channel Attack (SCA) and ultimately to obtain the keys.

During a SCA, physical information leaked during the operation of a cryptographic chip is collected and analysed by an attacker using a specific model and appropriate metrics to determine the key. This task can be transformed into a supervised classification task, so applying machine learning to SCAs is a natural fit. Deep learning is sensitive to data features and has the ability to learn and extract features intrinsic to the data. Deep Learning Side-Channel Attacks (DLSCAs), therefore, are more efficient and easier to implement than traditional side-channel attacks. Since 2016, several deep learning-based side-channel attack methods have been proposed.

Maghrebi et al. first applied deep learning techniques to SCAs in 2016 [4] and used multiple deep learning models for key attacks, achieving better results than traditional template attacks. In the literature [5], multi-segment leakage of power traces was exploited for joint attacks, improving the Convolutional Neural Network (CNN) performance of side channel attacks. Moonen et al. introduced heat map visualization techniques from the image processing domain to the DLSCAs domain to explore the effect of network depth on the effectiveness of side channel attacks [6]. In addition to this, there are several other works on deep learning side channel attacks, for example, literature [7] proposed a deep learning based side channel attack with time-frequency representation using CNN by exploiting time-frequency patterns and simultaneously extracting high level key correlation features in the spectrum; literature [8] showed by using multiple traditional deep learning models for side channel attack experiments that the side channel the impact of the diversity of target cryptographic chips in the attack on the experimental results. These research works show that deep learning-based side channel analysis performs well in terms of key attack efficiency and accuracy.

Currently, A fair proportion of of the existing DLSCAs are based on traditional neural networks. Improvement methods for the network are also achieved by increasing the width and depth of the network [7]. Although it can improve the accuracy of the recovery key, a large number of parameters need to be calculated during the training of the neural network, increasing the computational complexity of the algorithm. It also suffers from the problem of long training time and low training efficiency. Moreover, traditional neural networks do not make good use of features and have low quality of feature extraction. This paper proposes an improved lightweight convolutional neural network model based on Multi-scale Feature Fusion Networks (MFFN) [9]. Firstly, the features of the captured power consumption are extracted by convolutional kernels of different sizes. Different features of the power consumption under different sensory fields are captured and then feature fusion is performed. This results in richer data features and increased feature utilization. Secondly, the fused features are fed into the convolutional layer and dimensionality is reduced by limiting the number of channels. Finally, the fused feature size is further dimensionalised to prevent overfitting due to too many parameters. In addition, most of the current deep learning-based side channel attack models still use traditional evaluation metrics for analysis. In order to more accurately analyse the feature extraction capability of the model for power consumption, this study also introduces the Grade-Class Activation Map (Grad-CAM) [10], a weight visualisation method, combined with the Correlation Power Analysis (CPA) [11] method. The new method was used to carry out the analysis of model feature extraction capability based on heat map visualisation, and the feature extraction capability of different models was visually compared and analysed. The experimental results show that the multi-scale feature fusion convolutional neural network has better performance than the traditional neural network in terms of accuracy, efficiency and feature extraction effect.

## Background

### AES

Advanced Encryption Standard (AES) is a symmetric packet encryption algorithm widely used around the world [12], using the same key for encryption and decryption. Depending on the length of the key, AES is divided into AES-128, AES-192 and AES-256 encryption algorithms [13]. In this paper, AES-128 is used, the key length is set to 128 bits and a full key contains 16 subkeys. Thus, each subkey is a byte. The AES encryption process operates on a 4 x 4 matrix of bytes called “state”, whose initial value is a block of plaintext. There are ten rounds in the AES-128 encryption cycle, the first nine rounds consist of four steps: SubBytes, ShiftRows, MixColumns and AddRoundKey. The last round does not contain MixColumns, but only the other three steps.

The SubByte operation uses a lookup table called substitution box (S-Box) to achieve a byte-to-byte mapping and the result will be loaded onto the data bus. S-box substitution is the only non-linear transformation in the AES algorithm. At the same time, S-box substitution is a closely related operation to the key and is highly relevant to the security of the encryption algorithm. The attacker can reverse the key by the intermediate value (i.e. the value taken out of the S-box), and S-box substitution is used in a higher number of operations on the encryption device, and accordingly the power consumption generated by this operation is more obvious than the general operation, making it easier to find the location of this operation in the power traces to enhance the efficiency. Therefore, most of the side channel attacks, especially the power side channel attacks, choose the first round of S-box substitution or the last round of S-box substitution of AES algorithm as the attack point, and this paper is no exception. In our experiments, we focus on the first subkey k0 and others will be the same.

### Deep-leaning based side-channel attacks

Side channel attacks are based on analysing the data obtained from the device during the execution of the algorithm, rather than the algorithm itself. Such data is known as leakage, and the types of leakage include time delay, power consumption and electromagnetic radiation. Leakage information generated by the encryption device can be captured by an attacker using a device such as an oscilloscope. This information is then used to recover the keys associated with the encryption process.

The instantaneous power consumption of a cryptographic device depends on the data processed and the operations performed by the device [14]. Power consumption attacks exploit this fact to obtain keys by analysing the power consumption generated by the device during the encryption process. The attacker collects power consumption information and analyses it using a specific model and appropriate metrics to determine the key. When implementing a side-channel attack, the attacker first needs to model the power consumption of the cryptographic device when running the encryption algorithm. There are three energy models, namely the identity (ID), Hamming distance (HD) and Hamming weight (HW) models [8]. For the different power consumption models, the power consumption is labelled differently. In this paper, we choose the ID model, which corresponds to a total of 2^8 = 256 types of labels. This process can be transformed into a supervised classification problem. The task of classifying a given piece of data deep learning is fully applicable and effective pattern recognition and feature extraction algorithms can be constructed. So we combine deep learning with the following steps to perform a side-channel attack.

The premise of DLSCAs is that the attacker can control an analysis device that is similar to the victim device. Deep learning training is used as the analysis method. DLSCAs are divided into two phases.

In the analysis phase, the attacker selects a suitable attack point to obtain the power consumption. We choose the first round of S-box substitution of the AES as the attack point. We set the labels trained by each model in the experiment to the output state of the S-box byte substitution in the first round of cryptographic operations, denoted as

$$\begin{array}{c} {\text{state}}_{0}=S\text{-}box\left(p_{0}\right)⊕\left(k_{0}\right) \end{array}$$ (1)

In Eq (1), ‘⊕’ denotes a per-bit xor operation.*p*_0_ denotes the first byte of the plaintext. *k*_0_ denotes the first byte of the initial key. *state*_0_ denotes the state after the S-box output, which is the label. In order to explore the relationship between power traces and labels, a neural network model is built and trained. A well-trained network model during the attack phase can effectively classify power traces.

In the attack phase, the attacker uses the trained model to classify the traces collected from the victim device and derive the key from the obtained attack point’s value.

### Grad-CAM

Grad-CAM uses a two-dimensional fractional grid associated with the final output category of the network to compute elements at various locations of the input image. Grad-CAM shows which parts of the image ultimately play an important role in the selection of that output category, and Grad-CAM shows which locations of the image allow the neural network to make the final classification decision in the form of a heat map that can locate specific targets in the image. It is implemented by weighting each channel in this feature map with the gradient of the category relative to each channel, given an input image, for the output feature map of a convolutional layer.

In the Grad-CAM method, the feature activation map is obtained directly from the derivative of the feature map. Since the number of output channels of the last convolutional layer is set to be the number of categories to be classified, the derivative of the output feature map of the convolutional layer can be obtained directly from the category output results, which can be calculated to obtain one weight result for each category. The formula is as follows.

$$\begin{array}{c} \alpha_{p}^{c}=\overset{\text{global}\;\text{average}\;\text{pooling}}{\overset{︷}{\frac{1}{Z}\sum_{i}\sum_{j}}}\;\underset{\text{gradients}\;\text{via}\;\text{backprop}}{\underset{︸}{\frac{\partial y^{c}}{\partial A_{ij}^{p}}}} \end{array}$$ (2)

In Eq (2), $A_{ij}^{p}\in R^{u*v}$ is the kth feature map of width u and height v. *y*^*c*^ is the calculated classification result. $\alpha_{p}^{c}$ is a weighting result for each category. The Grad-CAM visualisation is also obtained by accumulating $\alpha_{p}^{c}$ with the feature maps. The weights indicate the importance of the target category c for the feature map. Since the focus is only on the influence of positive values in the feature map on the final classification result, it is necessary to remove the influence of negative values using a further ReLU function on the resulting feature map weighted by the weights [15]. The output map of Grad-CAM can be obtained after this weighting is done as follows:

$$\begin{array}{c} L_{\text{Grad-CAM}\;}^{c}=\text{ReLu}({\sum_{}}_{p}\alpha_{p}^{c}A^{p}) \end{array}$$ (3)

*L*_Grad-CAM_ in Eq (3) represents the weighted output of the feature map. Normalising it to between 0 and 1 draws a heat map superimposed on the original map, corresponding to the category region of interest in the image.

## DLSCAs based on multi-scale feature fusion networks

### Multi-scale Feature Fusion Networks (MFFN)

A traditional convolutional neural network consists of a convolutional layer, a pooling layer and a fully connected layer. The convolutional layer extracts the features of the target by layer-by-layer abstraction. Deep layer networks have a relatively large perceptual field [16] and a high ability to represent semantic information [17], but lack spatial geometric feature details due to low resolution, while shallow layer networks are the opposite.

**Fig 1:**
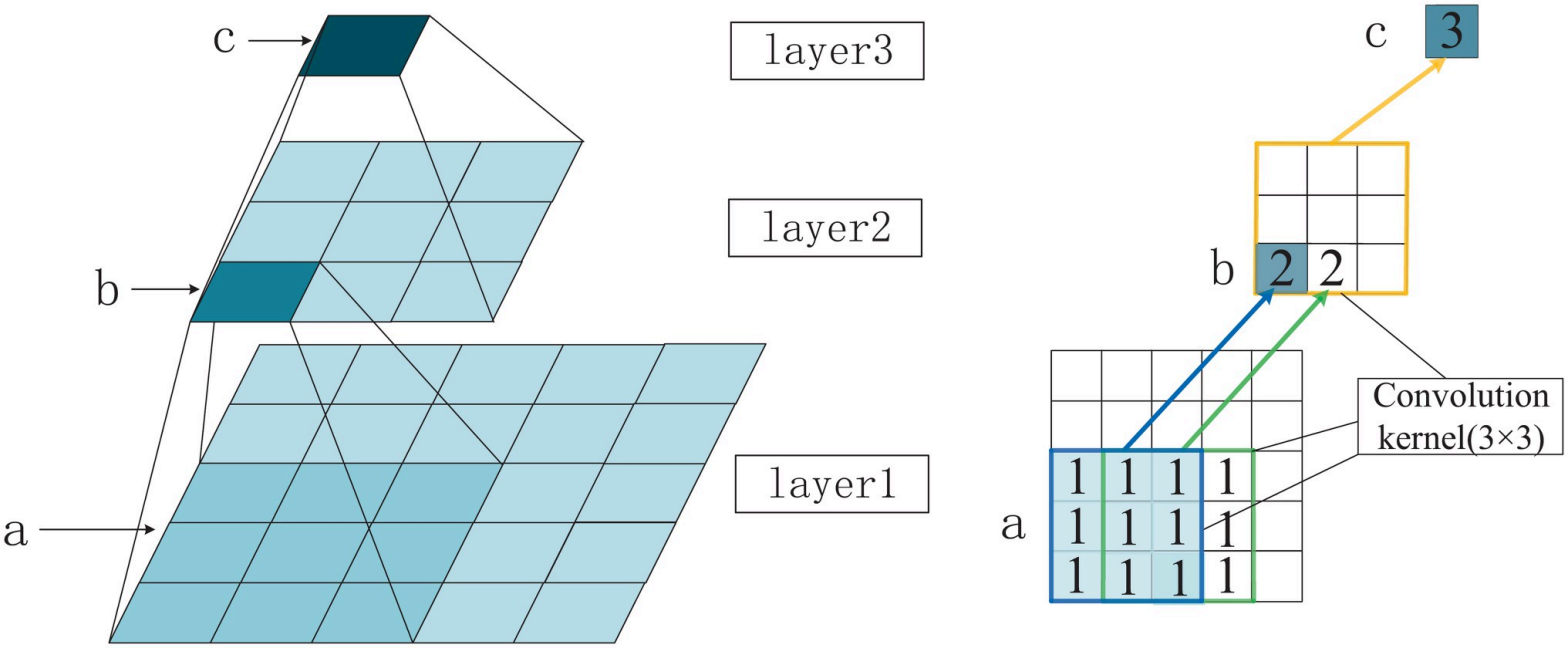
The receptive field of CNN.

Receptive Field refers to the size of the region of the output node on the feature map of each layer of the CNN corresponding to the mapping of the input of the previous layer. The feature vector of a point in the feature map is calculated from the input of the corresponding region in the previous layer. Other input values outside the region have no effect on this feature vector. This corresponding region is the perceptual field of this point. Fig 1 shows the receptive field of a convolutional neural network. From the bottom up (from shallow to deep) are layers 1, 2 and 3 respectively. The value of region b in layer 2, the eigenvector, is extracted from the values of region a of size 5 × 5 in layer 1 by a convolutional kernel of size 3 × 3. The receptive field of b is a. And so on, the unique value of the region in layer 3 is extracted from the values of all regions in layer 2 by a 3 × 3 convolution. layer 2 is the receptive field of c. The shallow network has a small field of sensation and a small down sampling factor, making it suitable for small targets. The deeper network has a larger field of sensation and a higher down sampling multiplier, making it suitable for detecting large targets. When detecting small targets, the deep layer feature map lacks the necessary resolution information, so it needs to be combined with the shallow layer information. In deep networks, after multiple downs ampling operations with layer-by-layer convolution, relatively much information is lost and detailed information of small targets may be lost in the process.

**Fig 2:**
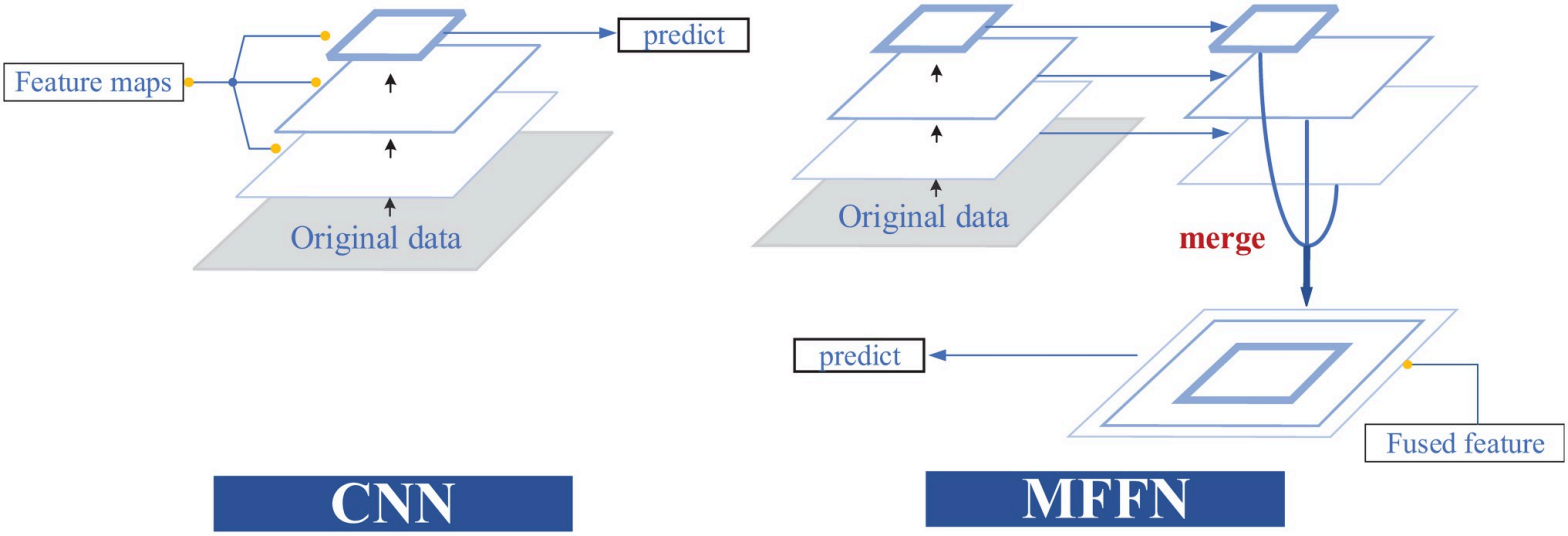
The integration of characteristics from deep and superficial layers of network.

Fig 2 illustrates the principle of deep and shallow multi-scale feature fusion. The diagram on the left shows the CNN network performing a layer-by-layer feature extraction operation on the raw data. From the bottom up the network gets progressively deeper and the receptive field gets progressively larger. Predictions are made using the features extracted at the deepest layer with the largest field of perception. The feature fusion network on the right, on the other hand, fuses the different features extracted from the deep and shallow layers using different scales of receptive fields, and finally uses the fused features for prediction. Traditional CNNs are inadequate for utilising features at different granularities. When using feature information from shallow networks for prediction, the network has low classification accuracy for small targets due to the lack of high-level semantic features. Predictions made by deeper networks lose detailed information. Multi scale refers to the sampling of input data at different granularities. The feature information extracted at different scales differs and has its own advantages, and we can use its features for different demanding tasks. However, MFFN can combine features extracted from different scale sensory fields to improve the utilization of data features and improve network performance.

**Fig 3:**
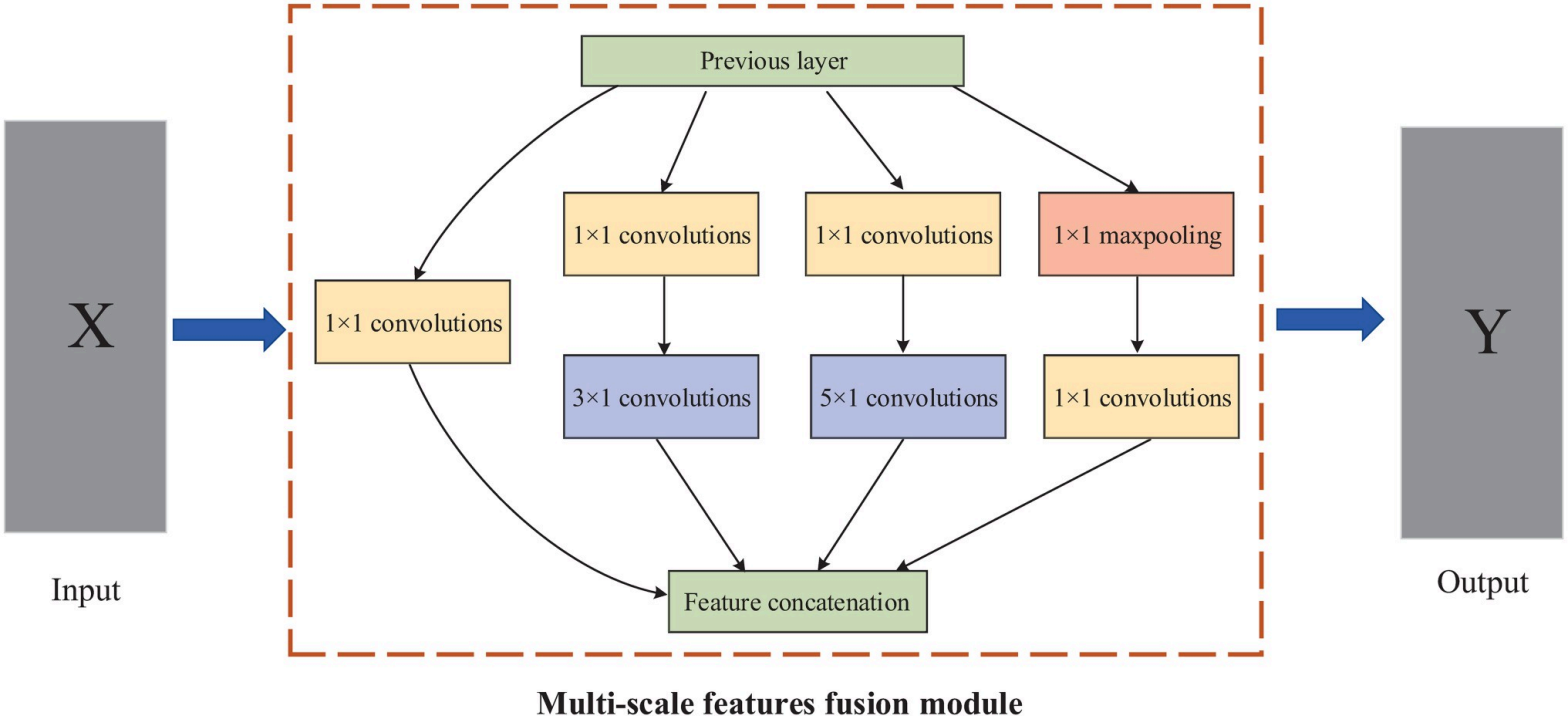
Multi-scale features fusion module.

In this paper, a lightweight Multi-scale Feature Fusion Convolution Neural Network (MFF-CNN) is constructed based on the traditional CNN, combined with the parallel multi-branch structure (inception module) of the feature fusion network [18]. Convolutional kernels of different sizes are used to capture features at different scales. As shown in Fig 3, the four parallel branching structures contain convolutional kernels of different sizes, namely 1 × 1 convolution, 3 × 1 convolution, and 5 × 1 convolution, combined with a 3 × 1 maximum pooling. The four channels are combined using concatenate column [19]. Features of different scales are fused in the third dimension channel of the data. A 1 × 1 convolution layer was also introduced to increase non-linearity and improve generalisation. The parallel multi-scale convolutional kernel is able to acquire features from different scales of the receptive fields at the same level, fuse them after unifying the dimensions and pass them to the lower layer of the network, facilitating the adjustment of the structure to balance the computational effort and model capability. MFF-CNN obtains fused features by combining the features extracted from the original data by the network through different scales of the receptive fields. The characteristics of the fused features are used to improve the utilization of the features and to achieve better results. The semantic information of the large receptive fields is used to help the network accurately detect or segment the target, while the representational power of the small receptive fields is used to provide detailed information. Combine the different characteristics of the multi-scale features input to the classification task in order to achieve improved classification accuracy.

### DLSCAs based on MFF-CNN

In traditional DLSCAs, a number of convolutional layers are applied when using CNNs to extract features from the input power traces by abstracting layer by layer. The features extracted by small-scale perceptual field convolution have a higher resolution and contain more location and detail information. However, the noise content is higher, the Signal-Noise Ratio (SNR) is lower and the semantics are weaker. There is less useful information for recovering keys which means the lack of highly data-dependent power consumption information. Features extracted by large scale perceptual field convolution have stronger semantic information, and the recovery key has more useful information, but not enough information about the details contained.

**Fig 4:**
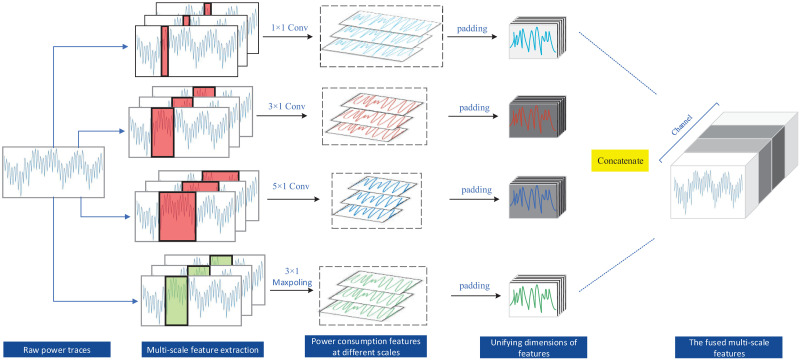
Extraction and fusion of multi-scale features.

By combining the multi-scale feature fusion module, the MFF-CNN model built in this paper can more accurately extract the temporal features of power consumption at different scales as well as power consumption features, and improve the feature utilization of power consumption. Fig 4 shows the basic process of feature extraction and fusion by MFF-CNN, where the original power traces data is extracted and fused with multi-scale features. The horizontal axis of the raw power traces image represents samples taken at a fixed sampling frequency over time, and the vertical axis represents the voltage data corresponding to the power consumption. For the power traces data as a one-dimensional timing signal, the n × 1 (n = 1, 3, 5) convolution is a non-linearly augmented time-series feature extractor at n granularity, which perceives each feature in the power consumption locally through filters at different scales, and then performs a synthesis operation on the local information at a higher level to obtain the global information after the extraction of different scales of sensory fields. A 3 × 1 pooling layer is also used for downsampling to retain salient features and reduce feature dimensionality. The dimensionality of the extracted feature vectors at different scales is integrated by the padding operation. The fusion of features from depth is carried out using the concatenate operation. The feature padding operation combines information from different channels to achieve cross-channel information interaction and integration, enhancing the interactivity between features at different scales. In a multi-scale feature fusion module, convolution and pooling operators of different dimensions are combined in parallel to improve the model’s ability to represent non-linear features and further learn the temporal sequence of power consumption and the internal correlation features of power consumption.

### Heat map visualisation of features

In this paper, MFF-CNNs are constructed based on traditional CNNs, using convolutional kernels of different scales to extract and fuse higher quality data features from raw power consumption trace data. Compared with traditional neural networks, MFF-CNNs possess better performance of automatic feature extraction and prediction. However, due to the black-box nature of neural networks, it is difficult to quantify the feature extraction capability of the network as it is not possible to understand the specifics of the internal feature data operation and influence decisions in the network layers. Therefore, a method based on the combination of CPA and heatmap visualization is proposed to analyze the feature extraction ability of the network.

We use Grad-CAM, a visualisation method, and apply it to a 1D time-series signal. The visual weight of each point of the power traces in the network is generated to judge the feature extraction capability of the network. Using a one-dimensional fractional grid associated with a particular output class, each sample point of the input power traces are calculated to obtain the importance of each sample point to the key classification decision. With Grad-CAM’s visualisation method, it is possible to understand which part of the power consumption made the network’s decision and to visualise which parts of the input traces had a positive impact on the final prediction.

Heat map visualisation is based on the idea of feature importance interpretation, looking for correlations between input variables, feature encoding and output results and presenting them visually in a visual display. However, the weight distribution of power consumption presented only by visualisation does not reflect the characteristics of the model, and not all points of the input trajectory are important for a neural network. Irrelevant points add noise and have a negative impact on the prediction. Therefore, networks that give more attention to the important parts of the power traces and less attention to the less important parts will perform better. We therefore combine correlation energy analysis to learn which points in the input power traces contain more information that an attacker can exploit. The power consumption SCA is based on the dependence of the power consumption of the cryptographic device on the operations performed and the data processed by the device. Thus the more data-dependent and operation-dependent sample points contain more information that is valuable for recovering the key. With CPA, we can filter these highly dependent sample points based on correlation. If these points show higher heat values in the traces coloured by the heat map, then these single points leaking informative power consumption have positively influenced the network in making the final decision. Then the network has extracted better and more valuable features. If points with lower relevance show lower heat values in the trajectory coloured by the heat map, then this indicates that power consumption with a high noise component and little available information has a low level of influence on the final decision and the network filters out redundant features. Therefore, the combination of heat map visualisation and correlation energy analysis allows the analysis and comparative evaluation of the feature extraction capability of the network.

Feature extraction effectiveness refers to the quality of the network in extracting features from a dataset. In this paper, we use a multi-scale feature fusion network to perform more efficient feature extraction on traces, and feature extraction effectiveness is an analytical tool that can be used to demonstrate the model’s ability to extract features. Combined with class activation graph visualisation, features in parametric form are mapped to human intuitively perceivable representations. A visual representation of the features learned by the network model for transmission and the correlation between inputs and outputs. The weighted heat map of network decisions is overlaid with the power traces to characterise the model and explain the decisions. It is shown which parts of the power traces play an important role in the final recovery key of the model. Combine this with correlation energy analysis to compare the heat value images of the power traces inside and outside the key interval where a single point leaks a large amount of information. The feature extraction capability of the model is evaluated in this way, with the simplicity of the process and the intuitiveness of the results.

The feature extraction capability of the network is demonstrated by:

1. The range of locations of the points within the interval of interest where the trace features contribute the most, and whether they overlap with the key interval of interest. This is expressed as the extent to which the range of the heat of the traces within the interval of interest above the horizontal value overlaps with the range of the key interval.
2. The high or low weight value of the points of the traces contributing features within the interval of interest. This is expressed as a high or low value of the heat value of the traces within the key interest interval, i.e. the degree of colour shading.

## Experimental setup

### Experimental equipment

**Fig 5:**
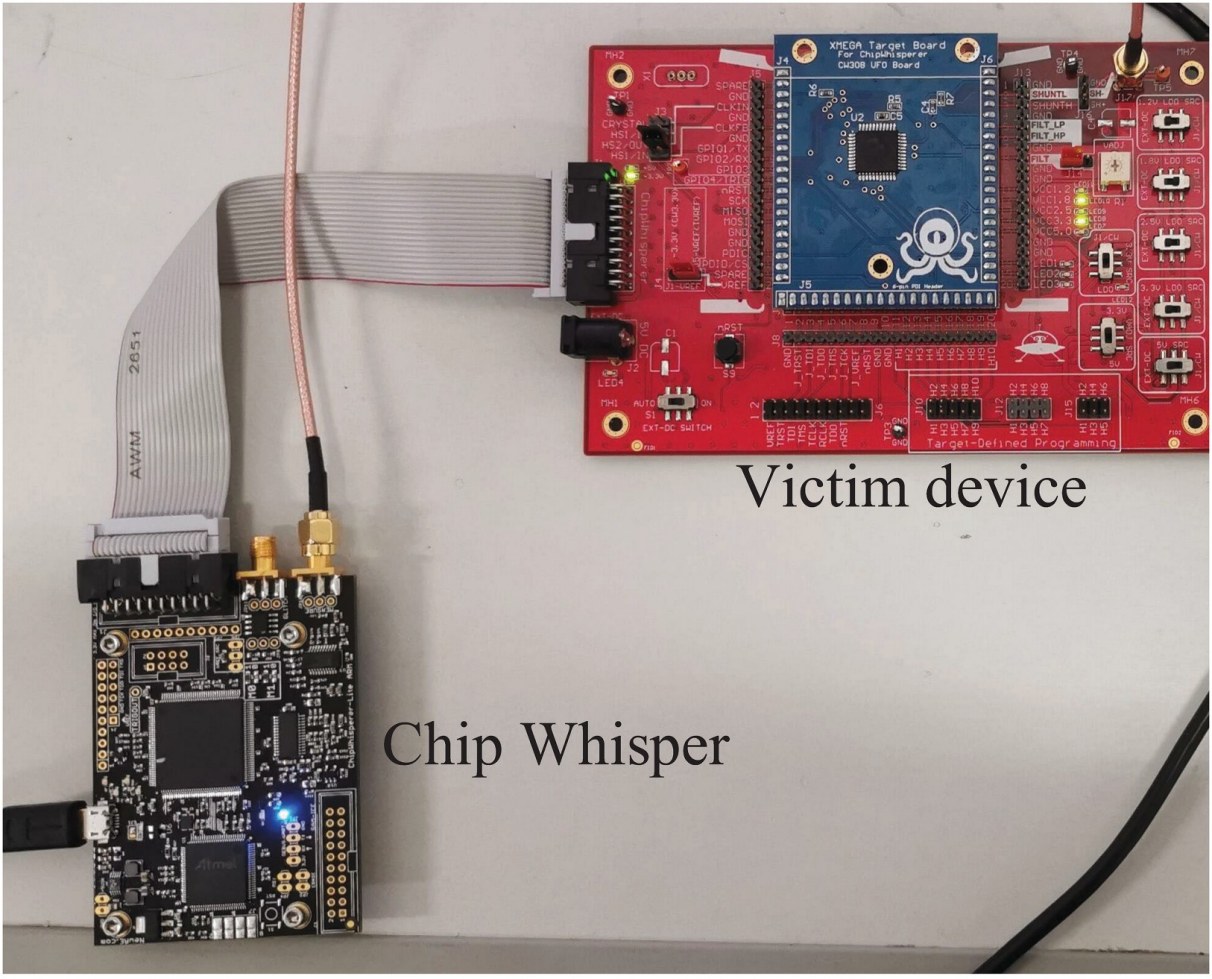
Experimental equipment.

All experiments in this paper were done in the same experimental environment configuration. For the experiments we used a ChipWhisperer [20] encryption device to capture power traces at a sampling frequency of 40MHz, using a target board, model CW308T-STM32F3, as shown in Fig 5. The encryption board is equipped with a 32-bit Arm Cortex-M4 encryption chip. The actual encryption algorithm running in the target encryption board is TinyAES-128C [21], and the encryption mode is that of an electrical code book (ECB). All models for this experiment were built and trained under the deep learning frameworks Keras-gpu 2.3.1 and tensorflow-gpu 2.1.0; the main hardware configuration of the computer was an Intel(R) Core(TM) i7-8400CPU @2.80GHz CPU and an NVIDIA GeForce GTX 3070 8GB GPU to perform all numerical calculations and model training in the experiments.

### Dataset

In this experiment, a total of 120,000 power traces were collected for the experimental dataset. We performed training data collection for the encryption board. The encryption device was encrypted using random plaintexts and random keys throughout the encryption process. 100,000 power traces were collected. After alignment, the first 90,000 of these were divided into a training dataset for the experiment and the last 10,000 were used as a validation dataset. Afterwards, test data were collected for the encryption plates. Random plaintexts and a fixed key were used for the encryption process. 20,000 power traces were collected. After the alignment process, they were used as the test data set for the experiments. Each power traces in the experiment contains 3,000 sampling points.

**Fig 6:**
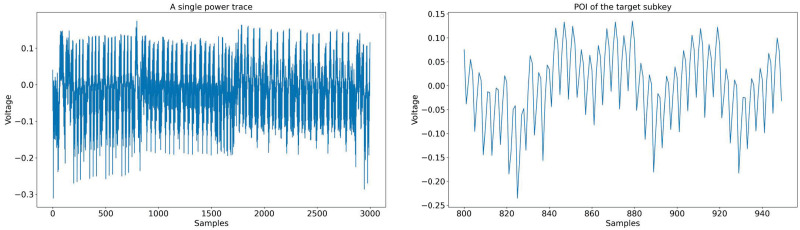
Raw power traces collected by encryption board. Left: A single power trace; rght: POI of the target subkey.

The encryption algorithm targeted in this paper is AES-128, and the target attack point chosen is the output location of the byte substitution in the first round of encryption operations of this encryption algorithm (i.e. the output of the S-box). The ultimate goal of the side channel attack experiment in this paper is to recover the first byte of the initial key block. Therefore, a CPA attack is performed on the power traces of the same key collected above, and the Pearson [22] correlation coefficient method is used to find the point in the trace with the highest correlation with the S-box output to find the interval of leaked information of the target byte, i.e. the points of interest(POIs). The interval identified above is used to feature select the power traces, and the data in the range is used as the feature vector to build the network model. The left part of Fig 6 shows the raw power traces taken from the encryption board. a shows the power information curve waveforms for all operations during the first round of encryption. This includes information on the round key addition, byte substitution, row shift and column obfuscation encryption operations. And the right part of Fig 6 shows the POIs for the target subkey.

### Evaluation metrics

(1) Model accuracy and loss
  Model accuracy refers to the probability of the model achieving correct classification results on the validation set [23]. In this experiment, the accuracy of the model is defined as.
  $$\begin{array}{c} \text{acc}\left({\text{X}}_{\text{attack}}\right)=\frac{\left|\left\{{\text{x}}_{\text{i}}\in {\text{X}}_{\text{attack}}\right\}\right|\tilde{\text{k}}|}{\left|{\text{X}}_{\text{attack}}\right|} \end{array}$$ (4)
  *X*_*attack*_ in Eq (4) represents the verification set data, xi represents the ith power trace in the dataset, and $\tilde{k}$ represents the correct key. Simplely, the accuracy of the model is also understood as the ratio of the number of power traces when the guessed key equals the correct key to the number of power traces in all verification sets.
  The loss of a model characterises the degree of deviation between the predicted and true values of the model. The smaller the loss value, the closer the model’s prediction is to the true value, the more reliable the prediction is and the more robust it is. Since the model experimented in this paper ends up with a multiclassification task, the loss value of the model is calculated using the categorical crossentropy loss function for multiclassification. And the weights and bias parameters of the model are optimized by back propagation.
(2) Model training time
  Training time is one of the most important criteria for evaluating the merits of a deep learning model. When two models are compared and the same accuracy is achieved, if the training time of a model is shorter, it indicates that the model performs better, the model converges faster and is easier to train for deep learning.

## Experiments

### Model training for MFF-CNN

**Fig 7:**
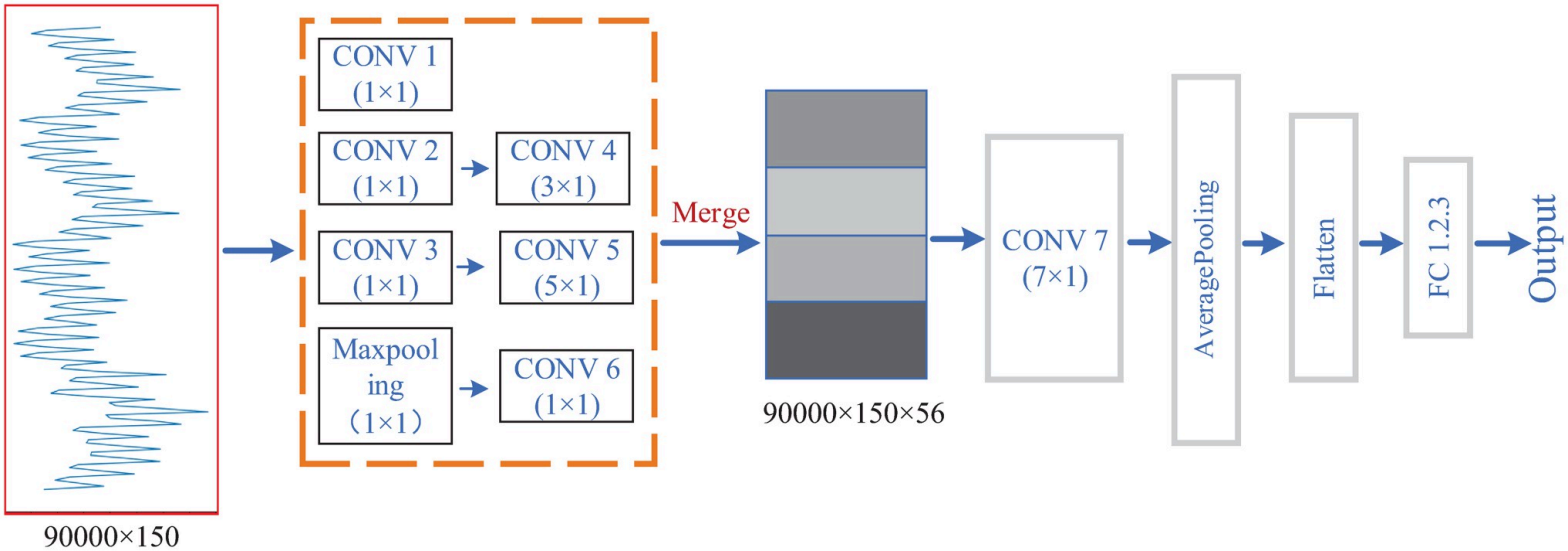
Side-channel attacks based on MFF-CNN.

The structural parameters of the MFF-CNN are shown in Fig 7 with Table 1. Since the segment in the trace which is related to the first S-Box operation contains 150 samples, so the input size of these models are set to 150. The output size of our models is defined by the data processed at the attack point. Since the data at that state is a byte, the output layers of the models contain 256 neurons for 256 classes. The first seven layers of the network are parallel multi-branch structures, using 1 × 1, 3 × 1, 5 × 1 convolutions and 1 × 1 maxpooling for feature extraction at different scales. The 1 × 1 convolution is added in front of the 3 × 1 convolution and the 5 × 1 convolution accordingly to aggregate the information and effectively reduce the number of parameters and increase the non-linearity. The above convolutions are followed by activation using the relu function. After obtaining multiple scales of power traces features, the features are synthesised by deep concatenation. The resulting fused multi-scale features are extracted by a layer of convolution and downsampled by an averaging pooling layer to remove redundant information and achieve the effect of downsampling and increasing non-linearity. The resulting 3D feature map is tiled into a one-dimensional vector using the flatten layer at layer 11 and then used as input to the fully-connected layer, which is finally used for the output of the softmax results. The model uses the RMSprop [24] optimizer and sets a learning rate of 0.0005 to ensure stable convergence.

**Table 1:** Structure parameters of MFF-CNN.

Layer Type	Output Shape	Parameter #
Input	(None, 150, 1)	0
Conv1D 1	(None, 150, 8)	16
Conv1D 2	(None, 150, 8)	16
Maxpooling1D	(None, 150, 1)	0
Conv1D 3	(None, 150, 8)	16
Conv1D 4	(None, 150, 16)	400
Conv1D 5	(None, 150, 16)	656
Conv1D 6	(None, 150, 16)	32
Concatenate	(None, 150, 56)	0
Conv1D 7	(None, 150, 2)	12576
AveragePooling1D	(None, 75, 32)	0
Flatten	(None, 2400)	0
Dense 1	(None, 128)	307328
Dense 2	(None, 256)	33024
Dense 3	(None, 256)	65792
Total Parameter:419,856		

### Comparative experiments

To investigate the effectiveness of MFF-CNN applications in side channel attacks, we conducted comparative experiments using several common deep learning models CNN, MLP and RNN. The CNN, MLP and RNN model structures used in references [25, 26], and other parameter settings are the same as those of MFF-CNN.

### Results and discussion

**Fig 8:**
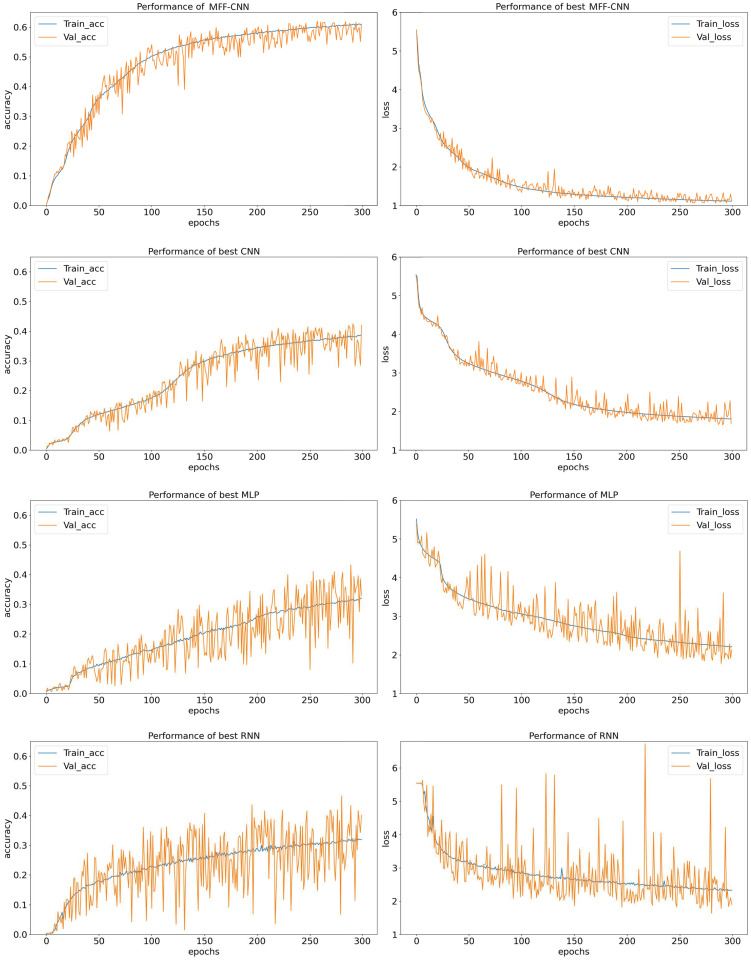
Training performance of the MFF-CNN,CNN,MLP and RNN.

Experiments are performed for the STM-32 dataset. In terms of accuracy and loss, side-channel attack efficiency, model stability and feature extraction effect, the side-channel attack performance of the model is comprehensively compared and analyzed. The experimental results are shown in Fig 8.

Side channel attack experiments were carried out for the 1st subkey of the key, both networks were tested in the same experimental environment, the same dataset was used for the experiments and each was trained for 300 epochs. Fig 8 shows the training performance of the four models. Among the three traditional neural network models, CNN, RNN and MLP, CNN outperforms RNN and MLP in all aspects. The accuracy growth converged only at the 150th epoch. Compared to CNN, MFF-CNN converges faster. In the first 80 epochs the accuracy of the model improved rapidly and converged, reaching an accuracy (Val_acc) of 55.04% on the validation set. At this point, the accuracy of the MFF-CNN with 80 epochs of training has surpassed that of all conventional networks with 300 epochs of training in the experiment. Table 2 shows the number of training sessions and time for each model when the accuracy of the validation set reaches 40%. When all models reach the same accuracy (40%) in the validation set, the shortest traditional network, CNN, requires 212 epochs and 5min42s of training time, while MFF-CNN requires only 44 epochs and 2min56s of training time, saving 51.46% of time compared to CNN, 61.40% of time compared to MLP and 95.18% less time than RNN. Table 3 shows the average classification accuracy and loss values of the four networks on the test set when all the optimal models were reached. 61.93% accuracy was achieved by the MFF-CNN, an improvement of 16.75%, 19.39% and 16.92% compared to the CNN, MLP and RNN respectively. Comparing the changes in the loss function, the CNN model has a tortuous and slow decreasing loss curve, and the loss curve on the validation set is more jittery and the model does not generalise well. In contrast, the MFF-CNN’s loss decreases rapidly in the first 50 epochs and converges to an optimum of 1.09 by stable convergence thereafter. Compared with CNN, MLP and RNN, the loss of MFF-CNN decreased by 0.50, 1.04 and 1.01 respectively. The validation set curve jitter was minimal and the generalization ability was good.

**Table 2:** Number of epochs and training time for each model at Val_acc = 40%.

	Epoch	Time for training
**MFF-CNN**	44	166s
**CNN**	212	342s
**MLP**	230	430s
**RNN**	107	3445s

**Table 3:** Average classification results of MFF-CNN,CNN,MLP and RNN.

	MFF-CNN	CNN	MLP	RNN
**Average accuray**	61.93%	46.18%	42.54%	45.01%
**Final loss value**	1.09	1.59	2.13	2.10

**Fig 9:**
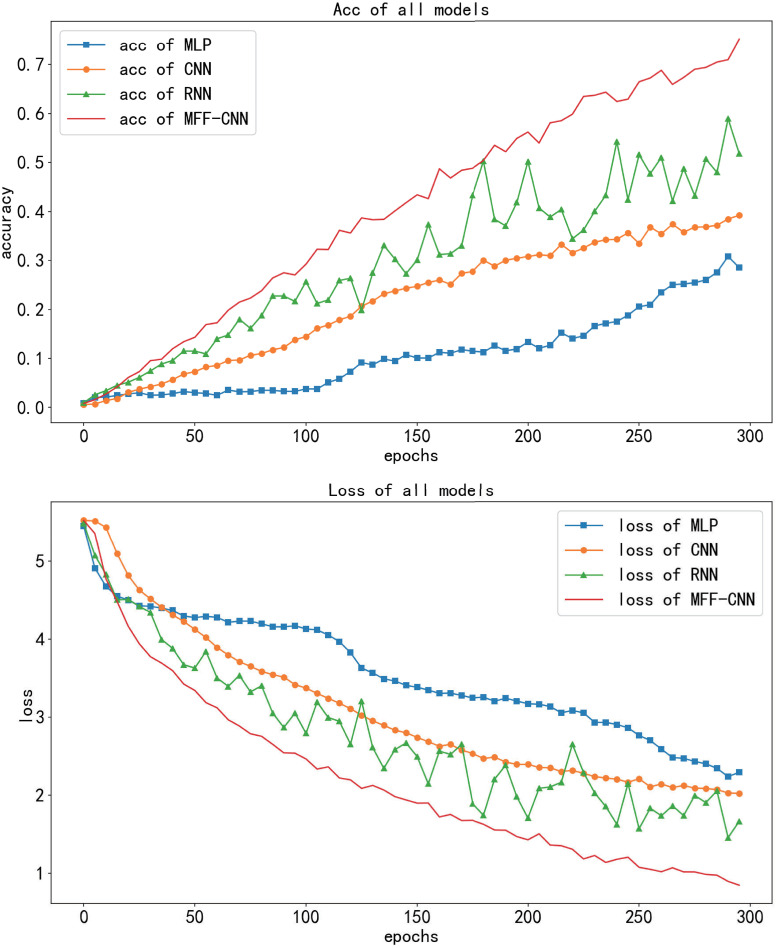
Training performance of the MFF-CNN,CNN,MLP and RNN (XMEGA).

In order to obtain reliable validation, we also conduct comparative experiments on the public dataset XMEGA [27]. A total of 6,000 power traces were used. 5,000 of them are divided into training datasets for experiments. 500 power traces as validation dataset. 500 power traces as validation dataset. The network structure parameter settings used in the experiment are the same as the experiment for STM32 data. The training process is shown in Fig 9. Similar to the experiments on the STM32 dataset, both acc and loss, MFF-CNN outperforms the three traditional neural networks. Table 4 shows the number of epochs and training time for each model at Val acc = 30%. MFF-CNN needs to train 103 epochs, and the training time is 24.31s, which saves 22.90% of the time compared to CNN and 95.22% of the time compared to RNN. The accuracy of mlp cannot reach 30.00%. Table 5 shows the average classification accuracy and loss values of the four networks on the test set when they all reach the optimal model. The accuracy of MFF-CNN reaches 61.93%. Compared with CNN, MLP and RNN, it is improved by 33.20%, 43.81% and 23.28% respectively. The loss of MFF-CNN is 0.95. Compared with CNN, MLP and RNN, the loss of MFF-CNN is reduced by 1.21, 1.68 and 1.15 respectively.

**Table 4:** Number of epochs and training time for each model at Val_acc = 30%(XMEGA).

	Epoch	Time for training
**MFF-CNN**	103	24.31s
**CNN**	261	32.91s
**MLP**	-	-
**RNN**	120	508.45s

**Table 5:** Average classification results of MFF-CNN,CNN,MLP and RNN(XMEGA).

	MFF-CNN	CNN	MLP	RNN
**Average accuray**	70.00%	36.80%	24.19%	46.72%
**Final loss value**	0.95	2.16	2.63	2.10

Through these comparisons, it can be obtained that the side channel attack efficiency and the performance of the optimal model of MFF-CNN are significantly better than those of traditional neural networks.

**Fig 10:**
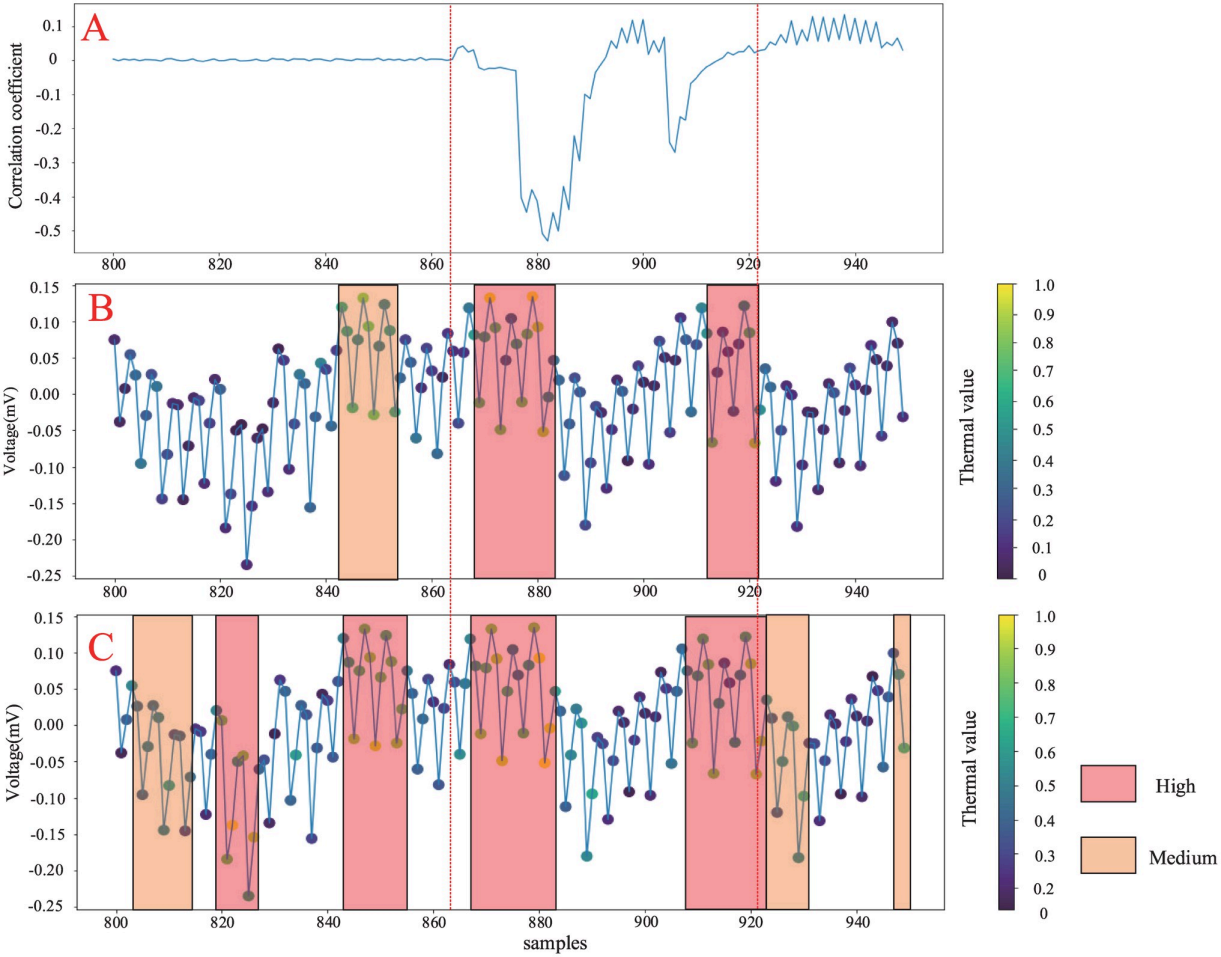
Comparison between FNN and CNN in correlation coefficient plot and weight visualisation trace within the interval of interest [800:950]. A: Correlation coefficient B: MFF-FNN weight visualization C: CNN weight visualization.

As the features of the power traces data are different from the image features, the thermal distribution of key features (e.g. geometric features, texture features) within the different thermal colouring regions cannot be observed as visually in the image. Therefore, we calculate linear correlation coefficients between the hypothetical power consumption and the measured energy traces in the interval of interest [800:950] in STM32 dataset to produce a correlation coefficient map, which is combined with the thermal map. This is used to provide a visual comparison of the feature extraction effect of the MFF-CNN and the best performing CNN model among the conventional models. Fig 10 shows the correlation coefficient plot of MFF-CNN, CNN within the interval of interest [800:950] compared to the weight visualization trace. With the correlation coefficient plot, it can be observed that the most substantial coefficient fluctuations exist within the interval [864:922], which is the point of interest for the attack. It is also the key interval in power traces. The interval contains important leaked information needed to perform a side-channel attack. The trace plot, visualised by colouring the heat map, shows how responsive the neural network is to each region and also shows where the features that contribute most to the model making the final decision are located.

The points in the MFF-CNN that contribute high feature weight values (influence coefficients above 0.8) are distributed in [868:882] and [912:922], both within the critical interval [864, 922]. In addition to the critical interval, a small number of points with medium feature weight values (thermal values 0.6 to 0.8) exist in the [842:852] interval. In the comparison performed on the CNN, it can be observed that the points with high values of contributing feature weights are distributed in [818:828], [844:855], [868:883] and [908:922] and are not concentrated within the key interval. The points with medium feature weight values are also scattered throughout the coloured trace at [803:815], [922:931] and [908:950]. All regions with medium CNN feature weights and a significant proportion of high weight regions, corresponding to an power consumption correlation coefficient of almost 0. Although we still extracted the main leakage through the CNN, the CNN network focused on the information in the key region of [864, 922]. However, information from non-critical regions was still extracted as high weighted features. The CNN did even 45.16% more feature extraction in the non-critical interval than in the critical interval. This indicates that the CNN extracts data features with very low relevance in the non-critical intervals. These data features contain little meaningful side channel information leakage and mainly contain noise components, but also have a significant impact on the final decision of the model. In contrast, the MFF-CNN filters useless and redundant features, and the extracted features are in the critical interval and contain the most gainful leakage information for CPA attacks. Through the above analysis, for the MFF-CNN network, its visual weight distribution implies that the part with the largest weight corresponds to the part with the highest correlation coefficient in CPA analysis. And CNN networks not only focus on the informative parts, but also learn the unimportant parts around the informative regions. Therefore, the model feature extraction capability is visualized. The high attention features in the key interval of CNN accounted for 40.00%. The key feature ratio of MFF-CNN is 66.67%. The improvement of 24.74% is obtained.

## Conclusion

In order to study the application of deep learning in side channel attack, this paper optimizes the convolutional operation and proposes a side channel attack method based on multi-scale feature fusion convolutional neural network. MFF-CNN can obtain the information of power traces features in different scale perceptual fields and improve the feature utilization by using different semantic information and feature details. The classification accuracy is improved by combining the power traces features at multiple scales of the classification task input. The experimental results show that the model of MFF-CNN has high accuracy and low loss. Compared with traditional neural networks, it has better results. For the STM32 dataset, MFF-CNN improves the accuracy by 16.75% and above. For the XMEGA dataset, MFF-CNN improves the accuracy by 23.28% and above. During the training of the model, the MFF-CNN did not show any overfitting phenomenon, and the convergence trend was stable and fast, with good robustness. The quality of feature extraction of the network was visualised and analysed by combining the results of the correlation energy analysis calculation with the heat map. The results demonstrate that the MFF-CNN can effectively filter information irrelevant to energy leakage and extract features that are more valuable for recovering keys than traditional neural networks. Therefore, the comprehensive performance of MFF-CNN-based side channel attacks is superior.

In future work, we will continue to investigate the application of deep learning techniques to side channel analysis and explore some signal processing methods. For example, we will explore how to optimize the network structure and explore the performance of MFF-CNN on different datasets in conjunction with heat maps.
